# Electrical Performance and Bias-Stress Stability of Amorphous InGaZnO Thin-Film Transistors with Buried-Channel Layers

**DOI:** 10.3390/mi10110779

**Published:** 2019-11-14

**Authors:** Ying Zhang, Haiting Xie, Chengyuan Dong

**Affiliations:** Department of Electronic Engineering, Shanghai Jiao Tong University, Shanghai 200240, China; Zhangying0036@sjtu.edu.cn (Y.Z.); haitingx@163.com (H.X.)

**Keywords:** amorphous InGaZnO, thin-film transistor, nitrogen-doping, buried-channel, stability

## Abstract

To improve the electrical performance and bias-stress stability of amorphous InGaZnO thin-film transistors (a-IGZO TFTs), we fabricated and characterized buried-channel devices with multiple-stacked channel layers, i.e., a nitrogen-doped a-IGZO film (front-channel layer), a conventional a-IGZO film (buried-channel layer), and a nitrogen-doped a-IGZO film (back-channel layer). The larger field-effect mobility (5.8 cm^2^V^−1^s^−1^), the smaller subthreshold swing value (0.8 V/dec, and the better stability (smaller threshold voltage shifts during bias-stress and light illumination tests) were obtained for the buried-channel device relative to the conventional a-IGZO TFT. The specially designed channel-layer structure resulted in multiple conduction channels and hence large field-effect mobility. The in situ nitrogen-doping caused reductions in both the front-channel interface trap density and the density of deep states in the bulk channel layers, leading to a small subthreshold swing value. The better stability properties may be related to both the reduced trap states by nitrogen-doping and the passivation effect of the nitrogen-doped a-IGZO films at the device back channels.

## 1. Introduction

Amorphous silicon thin-film transistors (a-Si TFTs) are the mainstream of active-matrix devices for flat panel displays (FPDs); additionally, polycrystalline (p-Si) TFTs are used to address some high-standard FPD products. In recent years, amorphous InGaZnO (a-IGZO) has been extensively studied as a potential material for the channel layers of TFT devices. In fact, a-IGZO TFTs are considered to replace silicon TFTs owing to their high mobility, low-temperature deposition, good large-area uniformity, and simple processing methods [1,2,3].

However, the electrical performance and stability properties of a-IGZO TFTs still need further improvements for their applications in FPDs and other fields [4,5]. Recently, some researchers—including our group—reported that nitrogen-doping (N-doping) effectively improved the electrical properties (e.g., subthreshold swing (SS) and bias-stress stability) of a-IGZO TFTs by decreasing the number of deep states and oxygen vacancies (Vo) in the device channel layers and reducing the channel/dielectric interface trap density with N atoms incorporated into the a-IGZO film [6,7,8]. However, the field-effect mobility (μ_FE_) of the nitrogen-doped a-IGZO (a-IGZO:N) TFT devices also decreases due to the suppression of the oxygen vacancy (Vo) level in their channel layers, the main source of free electrons in oxide semiconductors [9,10]. Therefore, N-doping is an effective method for improving the electrical properties of a-IGZO TFTs, but its shortcomings still need to be overcome.

In this study, buried-channel a-IGZO:N TFTs, i.e., devices with multiple-stacked channel layers, were fabricated and evaluated. These devices showed better performance and stability than the conventional devices. Specifically, the channel layer of the buried-channel device consisted of an a-IGZO:N layer (front-channel layer), an a-IGZO layer (buried-channel layer), and an a-IGZO:N layer (back-channel layer). We believe this device structure is a feasible approach to improving the electrical properties of a-IGZO TFTs.

## 2. Materials and Methods

**Figure 1:**
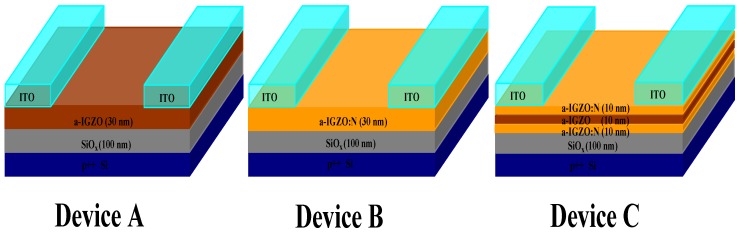
Schematic cross sections of the amorphous InGaZnO thin-film transistors (a-IGZO TFTs) prepared in this study.

Inverted staggered a-IGZO TFTs were prepared on p+ heavily doped silicon wafers with 100 nm thick thermal oxide (SiO_2_). The silicon wafers and thermal SiO_2_ were used as the gate electrodes and gate insulators of the TFT devices, respectively. For comparison, three types of TFT devices with different channel layers were fabricated, as shown in Figure 1. A 30 nm thick single channel layer was formed with conventional a-IGZO for Device A and with a-IGZO:N for Device B, respectively. Then, Device C with the buried-channel layer structure (10 nm thick a-IGZO:N (front-channel layer) + 10 nm thick a-IGZO (buried-channel layer) + 10 nm thick a-IGZO:N (back-channel layer)) was designed and prepared.

The channel layers and the source/drain (S/D) electrodes were deposited with the RF-magnetron sputtering technique. The sputtering chamber was evacuated to a base pressure (<3 × 10^−6^ Torr) before the film depositions. The gas pressure was fixed at 3 × 10^−3^ Torr during the sputtering process. The channel layers were prepared at room temperature (RT) using an IGZO target (In_2_O_3_:Ga_2_O_3_:ZnO = 1:1:1 mol%); the RF power and the Ar flow rate were 60 W and 10 sccm, respectively. As for the depositions of the a-IGZO:N films, the nitrogen gas (N_2_) was fed into the sputtering chamber at the flow rate of 1.2 sccm, and the Ar flow rate was fixed at 10 sccm. Then, the 100 nm thick indium-tin-oxide (ITO) layers were prepared as S/D electrodes in the same sputtering chamber. For simplicity, no passivation layers were prepared in this study. Both the channel layers and S/D electrodes were patterned by the shadow masks during sputtering; the channel width (*W*) and length (*L*) of the a-IGZO TFTs were fixed at 1000 and 250 μm, respectively. Finally, the TFT devices were annealed in N_2_ atmosphere at 380 °C for 1 h.

The electrical measurements for the a-IGZO TFTs were performed at RT using an electrical analyzer (Keithley 4200, Keithley, Cleveland, OH, USA). For the transfer curve tests, the drain-source voltage (*V*_DS_) was fixed at 10 V, and the gate-source voltage (*V*_GS_) ranged from −20 to 40 V.

## 3. Results and Discussion

**Figure 2:**
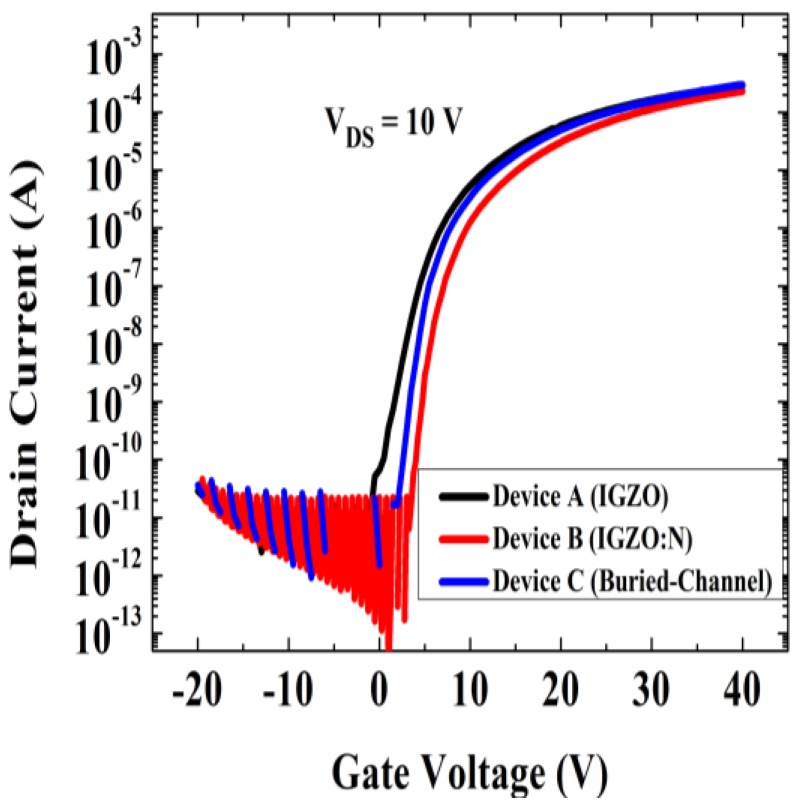
Transfer curves of the prepared TFTs (Devices A, B, and C).

Figure 2 and Table 1 show the transfer curves and the corresponding extracted electrical parameters of the TFT devices, respectively. Here the μ_FE_ was obtained graphically from the square root of drain current (*I*_DS_^1/2^) versus the gate voltage (*V*_GS_) in the saturation region using the intercept and maximum slope [11]. The threshold voltage (*V*_TH_) was extracted from the gate voltage value where *I*_DS_/(*W*/*L*) = 1 nA. The subthreshold swing (SS) was defined as the half value of the difference between the gate voltages corresponding to the drain currents of 10^−10^ A and 10^−8^ A. The on–off current ratio (*I*_ON_/*I*_OFF_) was obtained from the ratio of the maximum and minimum drain current values within the *V*_GS_ range of −20 to ~40 V.

As switching devices in FPDs, TFTs are expected to have high field-effect mobility and low subthreshold swing, which can lead to better switching speed and smaller power consumption. As shown in Figure 2 and Table 1, the a-IGZO:N TFT (Device B) showed an improved SS value (0.8 V/dec) but degraded μ_FE_ (4.7 cm^2^V^−1^s^−1^) compared with those of the conventional IGZO TFT (Device A, SS = 1.3 V/dec and μ_FE_ = 5.8 cm^2^V^−1^s^−1^). In fact, this result was consistent with the other reported a-IGZO:N TFTs [7,9]. Besides, from Figure 2 one may clearly observe that the N-doping process could cause a positive *V*_TH_ shift for a-IGZO TFTs. As shown in Table 1, the *V*_TH_ increased from 2.0 V (Device A) to 5.0 V (Device B) with the nitrogen doped into the channel layers of the a-IGZO TFT. This result also agreed well with the findings of a recent study of a-IGZO:N TFTs [12]. Most importantly, the buried-channel TFT (Device C) showed the best electrical performance in this study. As shown in Table 1, Device C exhibited the smallest SS value (0.8 V/dec) as well as the largest μ_FE_ (5.8 cm^2^V^−1^s^−1^) among all three devices. In addition, the *V*_TH_ of the buried-channel device (3.5 V) lay between those of the conventional a-IGZO TFT (Device A) and the a-IGZO:N TFT (Device B). It should be noted here that all the tested devices exhibited reasonably good *I*_ON_/I_OFF_ values (>10^8^), as shown in Figure 2 and Table 1.

**Figure 3:**
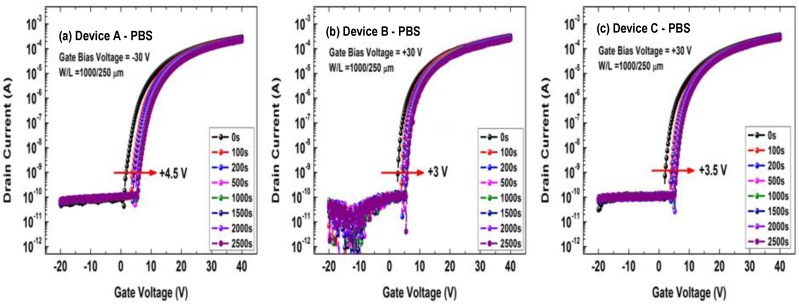
Positive bias stress (PBS) testing results of the a-IGZO TFTs: (**a**) the conventional a-IGZO TFT (Device A), (**b**) the a-IGZO:N TFT (Device B), and (**c**) the buried-channel TFT (Device C). The stressing conditions were *V*_GS_ = +30 V and *V*_DS_ = 0 V.

Figure 3 shows the transfer curves of the a-IGZO TFTs under positive bias stress (PBS) tests. For measurements, the gate electrodes were applied by +30 V with the drain and source grounded; after a period, the transfer curves were instantly measured (*V*_DS_ = 10 V). As shown in Figure 3, the transfer curves of the three devices shifted positively to different extents as the stressing time elapsed. As shown in Figure 3a, the positive *V*_TH_ shift of the conventional a-IGZO TFT (Device A) was as large as +4.5 V under 2500 s of PBS testing. In contrast, the a-IGZO:N TFT (Device B) showed a much smaller *V*_TH_ shift (+3 V) than that of the conventional a-IGZO TFT, as shown in Figure 3b. As for the buried-channel device (Device C), the maximum *V*_TH_ shift value (+3.5 V, as shown in Figure 3c) lay between those of Devices A and B. In fact, the PBS stability of Device C was quite close to that of Device B, as shown in Figure 3. In other words, during PBS tests, the buried-channel TFT (Device C) showed more stable properties than the conventional a-IGZO TFT (Device A).

**Figure 4:**
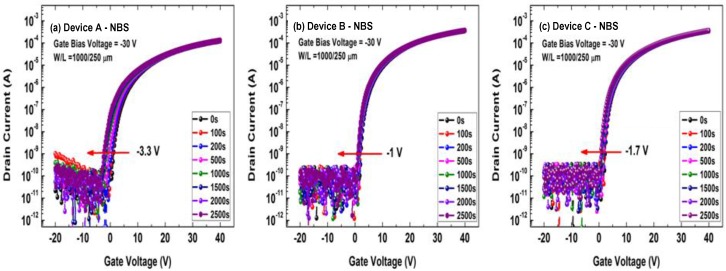
Negative bias stress (NBS) testing results of the a-IGZO TFTs: (**a**) the conventional a-IGZO TFT (Device A), (**b**) the a-IGZO:N TFT (Device B), and (**c**) the buried-channel TFT (Device C). The stressing conditions were *V*_GS_ = −30 V and *V*_DS_ = 0 V.

Figure 4 shows the transfer curves of the a-IGZO TFT devices under the negative bias stress (NBS) tests, where the stressing conditions were as follows: *V*_GS_ = −30 V and *V*_DS_ = 0 V. The NBS measurement operation was the same as that of PBS. One may observe from Figure 4 that the transfer curves of the three devices exhibited different negative shifts during NBS tests. As shown in Figure 4a,b, the maximum *V*_TH_ shift of the conventional a-IGZO TFT (−3.3 V) was much larger than that of the a-IGZO:N TFT (−1 V). Importantly, Device C showed a similar *V*_TH_ shift (−1.7 V after 2500 s of NBS testing) to that of Device B, implying that the buried-channel device (Device C) also exhibited better stability than the conventional a-IGZO TFT (Device A) during NBS tests.

**Figure 5:**
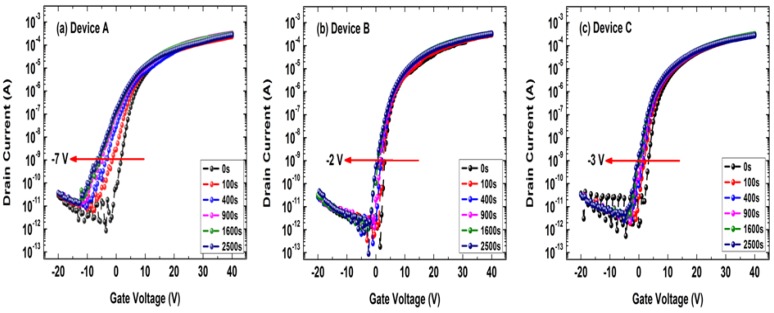
Negative bias stress with ultraviolet (UV) light illumination (NBIS) testing results of the a-IGZO TFTs: (**a**) the conventional a-IGZO TFT (Device A), (**b**) the a-IGZO:N TFT (Device B), and (**c**) the buried-channel TFT (Device C). The stressing voltage was *V*_GS_ = −20 V; the wavelength and power intensity of the UV light used in this test were 380 nm and 0.1 mW/cm^2^, respectively.

Figure 5 shows the transfer curves of the a-IGZO TFT devices under negative bias stress with ultraviolet (UV) light illumination (NBIS) tests. The stressing voltage was *V*_GS_ = −20 V; the wavelength and power intensity of the UV light were 380 nm and 0.1 mW/cm^2^, respectively. Both the light illumination and gate stressing were applied for a period, and then the transfer curves were immediately measured (*V*_DS_ = 10 V). According to Figure 5, one may notice that all three devices exhibited serious negative shifts during NBIS tests. As shown in Figure 5a, the maximum *V*_TH_ shift of the conventional a-IGZO TFT (Device A) was up to −7 V, which was the worst among all the tested samples. However, this negative *V*_TH_ shift could be effectively reduced to −3 V using the buried-channel structure (as shown in Figure 5c) or to −2 V by adopting the N-doping technology (as shown in Figure 5b). Apparently, the NBIS stability of Device C was more similar to that of Device B than that of Device A. In other words, the experimental results proved that the buried-channel device (Device C) could lead to a significant improvement in NBIS stability compared with the conventional a-IGZO TFT (Device A).

It has been reported by our group and other researchers that the field-effect mobilities of oxide semiconductor TFTs were evidently improved by employing the double-stacked channel layers (DSCL) with a high defect-density channel layer and a low defect-density channel layer [13,14,15]. In this study, we further designed and prepared the buried-channel a-IGZO:N TFTs using the multiple-stacked channel layers composed of a 10 nm thick a-IGZO:N layer (front-channel layer), a 10 nm thick a-IGZO layer (buried-channel layer), and a 10 nm thick a-IGZO:N layer (back-channel layer). This design exhibited the best electrical performance (e.g., the smallest SS value, the largest μ_FE_, and the optimum *V*_TH_), as shown in Table 1. Furthermore, the buried-channel device exhibited similar stable properties to those of the a-IGZO:N TFT during PBS, NBS, and NBIS tests, which were much better than those of the conventional a-IGZO TFT. The related physical mechanisms could be ascertained by analyzing the channel-layer structure of these devices.

**Figure 6:**
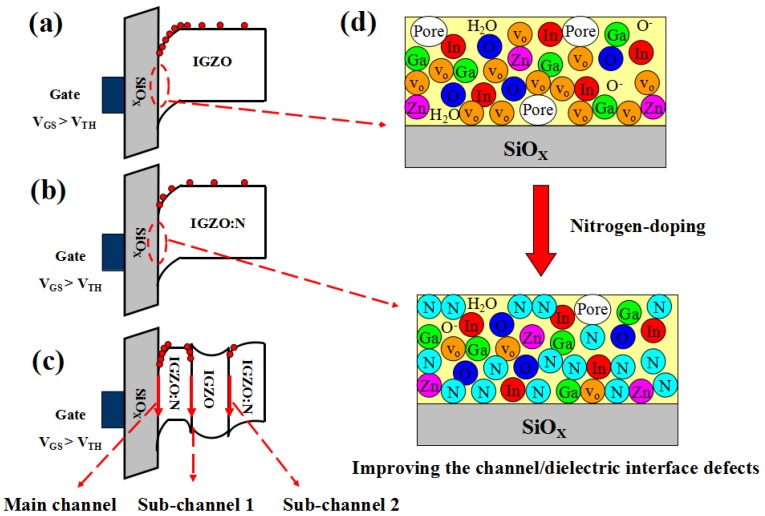
Energy band diagrams for (**a**) the conventional a-IGZO TFT (Device A), (**b**) the a-IGZO:N TFT (Device B), and (**c**) the buried-channel a-IGZO TFT (Device C). (**d**) The schematic illustration of the improvement effect for the N-doping process on the trap states in the bulk-layers and interfaces.

Figure 6 shows the energy band diagrams for Devices A, B, and C. As reported previously [7,9], N-doping caused a mobility reduction due to the significant suppression of the Vo level in the bulk channel layer. Former studies [15,16,17] indicated that N-doping reduced the Vo and defect density in the a-IGZO films. Hence, there was more Vo acting as the origin of free carriers in the conventional a-IGZO layer compared with that in the a-IGZO:N film. Therefore, one could assume that fewer free electrons took part in conductivity for Device B compared with the case for Device A (as shown in Figure 6a,b). This resulted in a lower mobility for Device B.

But, why did the buried-channel device not show degraded mobility as the a-IGZO:N TFT did? We attributed this fact to the particular energy band structure of the buried-channel device, as shown in Figure 6c. The electrons tended to inject from the conventional a-IGZO layer towards both the bottom a-IGZO:N layer and the top a-IGZO:N layer owing to the electron concentration difference between the conventional a-IGZO layer and the a-IGZO:N layer. Due to the positive polarity of the a-IGZO layer, the electrons accumulated in both the bottom a-IGZO:N/a-IGZO interface and the top a-IGZO/a-IGZO:N interface. During the operation of the buried-channel TFT device, these accumulated electrons also took part in conductivity, forming a first sub-channel at the bottom a-IGZO:N/a-IGZO interface and a second sub-channel at the top a-IGZO/a-IGZO:N interface. In other words, the conduction current in the buried-channel TFT was not only dominated by the main channel (channel layer/dielectric layer interface), but also the two additional sub-channels (bottom a-IGZO:N/a-IGZO interface and top a-IGZO/a-IGZO:N interface). The total carrier concentration from these three conduction channels in the buried-channel device might be comparable only with that from the front channel in the conventional a-IGZO TFT, as shown in Figure 6a,c, leading to the μ_FE_ values of the buried-channel device (Device C) that were nearly equal to those of the conventional a-IGZO TFT (Device A). In fact, these qualitative analyses might also be confirmed by numerical simulations, which are commonly used in explaining and discussing electrical properties of semiconductor devices [18,19]. The related study is still ongoing.

As mentioned before, N-doping effectively decreased the interface trap density as well as the deep defect density in the channel layer by incorporating the N atoms into the a-IGZO film (as shown in Figure 6d). Since the SS values of TFTs are closely related to the channel/dielectric trap density and the number of deep states in the channel layers [7,9,20], it is quite reasonable that the a-IGZO:N TFT had smaller SS values than the conventional a-IGZO TFT. As shown in Figure 6b,c, both the front-channel interface and the nearby channel layer of the buried-channel device were the same as those of the a-IGZO:N TFT, which naturally led to the same SS values for both cases.

The evident stability improvements (during PBS, NBS, and NBIS tests) of the a-IGZO:N TFT with respect to the conventional a-IGZO TFTs were assumed to result from the following reasons. First, the incorporation of N atoms into the a-IGZO film led to the suppression of trap states in the bulk channel layer as well as those in the front-channel interface (as shown in Figure 6d). Second, the nitrogen atoms doped at the back channel might act as a passivation layer, which could effectively protect the device from the influence of ambient gas (such as O_2_, moisture, etc.). Third, the back-channel a-IGZO:N layer might shield the channel from UV light influence to a certain extent. As for the buried-channel device, the aforementioned stability-improving effects were also valid. However, the existence of the buried-channel layer (a-IGZO) more or less increased the trap states in the bulk channel layers compared with the a-IGZO:N TFT (Device B). Therefore, the stability properties (PBS, NBS, and NBIS) of the buried-channel device (Device C) were a little worse than those of the a-IGZO:N TFT (Device B), but still much better than those of the conventional a-IGZO TFT (Device A).

So far, we can make some overall comments on the buried-channel TFT devices. Compared with the conventional a-IGZO TFT, the buried-channel device exhibited better electrical performance (smaller SS value and non-degraded μ_FE_) and more stable properties (much smaller *V*_TH_ shifts during PBS, NBS, and NBIS tests). Compared with the a-IGZO:N TFT, the buried-channel device showed a little worse stability under PBS, NBS, and NBIS tests, but better electrical properties (larger μ_FE_ and the same SS value). Notably, the buried-channel structure could be easily achieved by one-pump-down depositions in the sputtering machines. Therefore, we believe the use of buried-channel devices is a feasible approach for improving the electrical performance and stability properties of amorphous oxide thin-film transistors.

## 4. Conclusions

Buried-channel TFTs with multiple-stacked channel layers including an a-IGZO:N film (front-channel layer), an a-IGZO film (buried-channel layer), and an a-IGZO:N film (back-channel layer) were fabricated and measured in this work. Compared with those of the conventional a-IGZO TFT, the better electrical performance (e.g., smaller SS value, larger μ_FE_, and optimum *V*_TH_) as well as the improved stability properties (e.g., smaller *V*_TH_ shifts during PBS, NBS, and NBIS tests) were obtained for the buried-channel device. The a-IGZO:N film used as the front-channel layer in the buried-channel structure could reduce both the interface trap density and the bulk-layer deep state density, leading to a smaller SS value (0.8 V/dec) in comparison with the conventional a-IGZO TFT device (SS = 1.3 V/dec). The non-degraded μ_FE_ (5.8 cm^2^V^−1^s^−1^) for the buried-channel TFT might be attributed to its three conduction channels, including the main channel at the interface between the channel layer and the dielectric layer, the first sub-channel formed at the bottom a-IGZO:N/a-IGZO interface, and the second sub-channel formed at the top a-IGZO/a-IGZO:N interface. In addition, the improved bias-stress and NBIS stability of the buried-channel TFT compared with those of the conventional a-IGZO TFT might be mainly due to the suppression of the trap states in the bulk channel layers, the reduction in the defects at the front-channel interface, and the passivation effect created by using the a-IGZO:N film as the back-channel layer.

## Figures and Tables

**Table 1:** Extracted electrical parameters of the amorphous InGaZnO thin film transistors (a-IGZO TFTs).

Device	A	B	C
μ_FE_ (cm^2^V^−1^s^−1^)	5.8	4.7	5.8
SS (V/dec)	1.3	0.8	0.8
*V*_TH_ (V)	2.0	5.0	3.5
*I*_ON_/*I*_OFF_	2.2 × 10^8^	3.4 × 10^8^	5.6 × 10^8^
